# The role of bone morphogenetic proteins in ankylosing spondylitis

**DOI:** 10.1186/ar3723

**Published:** 2012-03-08

**Authors:** Rik J Lories

**Affiliations:** 1 Division of Rheumatology, Katholieke Universiteit Leuven, Leuven, Belgium

## 

Bone morphogenetic proteins (BMPs) are members of the transforming growth factor-beta superfamily and were originally identified as proteins that induce a full cascade of endochondral bone formation in vivo and ectopically. We hypothesized that the BMP signaling pathway could also play a role in the process of ankylosis that characterizes ankylosing spondylitis and related spondyloarthritides. By over expression of noggin, a BMP antagonist, in a dedicated mouse model of joint ankylosis, we provided evidence for this hypothesis. Our current studies focus on the relationship between stromal cell activation and inflammation, a link that is essential in ankylosing spondylitis. Emerging functional and genetic evidence further corroborates the essential role of BMPs in these processes. BMPs can trigger different downstream signaling cascades, in particular Smad and p38 signaling. Inhibition of p38 signaling in vivo in the spontaneous ankylosing enthesitis model appears to accelerate ankylosis while inhibiting in vitro chondrogenesis. Genetic analysis of intercrosses between the susceptible DBA/1 strain and the H2 identical Balb/c strain point towards a region containing the BMP type Ib receptor as a factor determining genetic susceptibility. Recent clinical data suggest that levels of BMPs are higher in patients with progressive ankylosis in comparison with patients that show no structural progression of disease. Taken together, these data support our hypothesis that BMP signaling is a therapeutic target in ankylosing spondylitis.

